# Knee extensor training in patients with patellofemoral pain: a systematic review and synthesis

**DOI:** 10.3389/fresc.2025.1641054

**Published:** 2025-08-11

**Authors:** Ted Gunhamn, Haris Pojskic, Sofia Ryman Augustsson

**Affiliations:** Department of Sport Science, Faculty of Social Sciences, Linnaeus University, Kalmar, Sweden

**Keywords:** rehabilitation, physical therapy, resistance training, quadriceps, range of motion

## Abstract

**Objective:**

This systematic review and synthesis aimed to describe the frequency and characteristics of knee extensor exercise prescriptions within patellofemoral pain (PFP) interventions and to assess the extent to which key training variables are reported. By doing so, it sought to inform and support more transparent and standardized reporting practices in exercise-based rehabilitation for individuals with PFP.

**Method:**

This systematic review was conducted following PRISMA guidelines. A literature search was performed in January 2024 across Web of Science, PubMed, Scopus, CINAHL, and SportDiscus. Studies were included if they investigated interventions incorporating knee extensor exercises for PFP.

**Results:**

Seventy-nine studies met the inclusion criteria. The most commonly prescribed exercises were the straight leg raise, squat, and open-chain knee extension, typically performed as three sets of ten repetitions with bodyweight resistance. However, key training variables such as range of motion and intensity were often inadequately reported, limiting reproducibility and clinical applicability.

**Conclusion:**

Knee extensor training for PFP predominantly consists of low-load, moderate-volume exercises, differing from conventional strength training recommendations. The lack of detailed reporting on critical variables, such as intensity and range of motion, reduces the clarity and applicability of rehabilitation protocols. Standardized reporting and further research are needed to optimize exercise prescription for PFP management.

## Introduction

Patellofemoral pain (PFP) is one of the most common knee disorders in adolescents and younger adults (1, 2). PFP has a prevalence between 8% and 25% depending on the population and is usually twice as common in females (3). Individuals diagnosed with PFP often have activity-induced pain around or inside the kneecap, which limits their participating in physical activities and sports (4). A reduced muscle strength of the knee extensors is often seen in these individuals, but it is discussed whether this is a cause of the disorder or a consequence of it (5, 6). The etiology of PFP is still unknown but has been suggested to be multifactorial. Factors such as patellar maltracking, excessive patellofemoral joint pressure, reduction in muscle strength of the hip and knee extensors and imbalances of activation patterns of the M. Quadriceps seem to cause the problem (1, 7).

Despite the high prevalence of PFP, consensus on the most effective treatment remains limited. Current evidence suggests that exercise therapy targeting the knee, hip, and core muscles may be beneficial (1, 8). Among these, hip and core exercises are frequently used in rehabilitation because they typically avoid directly loading the knee joint and thus pose minimal risk of aggravating symptoms (9). In contrast, prescribing knee extensor training presents a more complex clinical challenge. Quadriceps weakness is a common and clinically significant impairment in individuals with PFP, closely associated with abnormal joint mechanics, movement dysfunction, and persistent pain (5, 6). Strengthening the knee extensors is therefore essential for restoring joint function and improving long-term outcomes. However, because such exercises inherently involve loading the patellofemoral joint, they carry a higher risk of inducing pain, creating a tension between therapeutic necessity and symptom management (1, 7, 8).

Adding to this complexity, the recommended treatment “exercise therapy” can be perceives as a broad and somewhat vague term, requiring practitioners to navigate through a wide range of training variables (10). Within the scope of exercise therapy, various variables can be adjusted, such as type of exercise, range of motion (ROM), training volume (sets and repetitions), and external load (intensity) (11). The manipulation of these factors significantly influences outcomes related to muscle strength gains and hypertrophy (11). However, increased ROM and intensity in patients with PFP appear to negatively affect the risk of pain due to increased forces in the patellofemoral joint (12–15).

While previous systematic reviews have highlighted the benefits of knee extensor strengthening in PFP rehabilitation, indicating that this training may significantly enhance both short- and long-term rehabilitation outcomes (8, 16), there remains limited guidance on how to prescribe these exercises in practice. Specifically, reporting on key training parameters such as intensity, volume, and ROM is often inconsistent or insufficient, making it difficult for clinicians to apply existing evidence with precision. This lack of standardization also limits the ability to compare interventions across studies, replicate effective protocols, or understand common clinical practices. As a result, translating general recommendations into structured, individualized exercise programs can be difficult. More consistent and detailed reporting of exercise prescriptions is needed to support evidence-informed, patient-specific rehabilitation strategies.

Given these challenges, there is a clear need to investigate how knee extensor training is currently utilized and prescribed in the management of PFP. This synthesis aims to describe the characteristics and frequency of knee extensor exercise prescriptions within PFP interventions and to assess the extent to which key training variables are reported. By doing so, it seeks to provide clinicians with clearer insights to inform evidence-aligned decision-making in the management of PFP and support more transparent and standardized reporting in exercise-based rehabilitation.

## Methods

### Study selection and eligibility criteria

The review was conducted and reported following the guidelines of the Preferred Reporting Items for Systematic Reviews and Meta-Analyses (PRISMA) and in accordance with the guidelines for implementing PRISMA in Exercise, Rehabilitation, Sport medicine and SporTs science (17, 18). The inclusion criteria were: (a) Full text, original and peer review articles published up to and the date the search was conducted and (b) exercise interventions, both random and non-random as well as controlled and non-controlled, which included training of the knee extensor muscles for treating PFP. Interventions could include other forms of treatment besides knee extensor training.

Exclusion criteria were: (a) article not available in English, (b) interventions that did not include exercises for the knee extensors muscles, (c) intervention with other diagnoses than PFP and (d) if the intervention had not yet been taken place, such as protocol papers.

### Search strategy and review process

To find relevant literature an electronic search was initiated and finished in the end of January of 2024 in the following five databases: Web of Science, Pubmed, Scopus, CINAHL and SportDiscus. The following search string (with variation depending on the database) was used: Patellofemoral joint pain OR patellofemoral pain syndrome OR anterior knee pain OR OR plica syndrome AND resistance training OR knee musc* OR knee stren* OR knee exerc* OR knee resis* OR quadriceps resis* OR quadriceps exerc* OR quadriceps stren* OR quadriceps musc*. The entire search with queries in the different databases can be found in Supplementary S1.

The first author (TG) exported all identified studies to the reference manager system Endnote (EndNote, Version EndNote20, Philadelphia, PA) where duplicates were removed. The remaining studies were screened by title and non-relevant were removed (e.g., not exercise intervention, no PFP) by the first author. The abstracts of the remaining studies were then read independently by the three authors to further screen for relevant studies and remove non-relevant studies (e.g., no knee joint exercise, protocols). The relevant studies were then compared and discussed to reach consensus of studies eligible for this systematic review.

### Data extraction

Following final selection, the first author independently extracted data by thoroughly reviewing all included articles and entering relevant information into a standardized Excel spreadsheet, which was collaboratively developed by all three authors. The following data items were identified and extracted from the included studies: (a) exercises targeting the knee extensor muscles—defined as any exercise that primarily involves activation of the quadriceps muscle group, including open and closed chain exercises as well as all types of muscle activation (concentric, eccentric, isometric); (b) number of repetitions and sets; (c) exercise intensity, classified as reported in the study (d) ROM during the exercise, if specified; (e) whether and how exercise progression was implemented, with progression recorded only when explicitly reported (e.g., increase in load, sets/reps, or complexity); (f) duration and training frequency of the intervention (e.g., sessions per week and overall intervention length); (g) type of training, categorized by modality (e.g., resistance training, neuromuscular training, functional strengthening); and (h) outcome measures used in each study. All classifications and definitions were applied consistently during data extraction, based on a predefined extraction template developed prior to the review.

Due to the considerable variation in factors such as duration, number of exercises employed, total training volume, and the possible inclusion of additional interventions alongside knee extensor training in the studies, a synthesis of outcome effects such as a meta-analysis was not pursued.

### Risk of bias

We evaluated the risk of bias in randomised clinical trials using the Revised Cochrane Risk-of-Bias Tool for Randomised Trials (RoB2) (19). RoB2 assesses bias across five key domains: the randomisation process, deviations from intended interventions, missing outcome data, outcome measurement, and selection of reported results. We categorised studies as having low, some, or high risk of bias: *low risk* if there was low risk in all five domains, *some risk* if there was some risk in at least one domain but no high risk in any domain, and *high risk* if there was high risk in one or more domains.

For non-randomised trials, we used the Risk Of Bias In Non-randomised Studies of Interventions (ROBINS-I) tool, to assess controlled intervention studies (20). ROBINS-I includes a seven-domain checklist to evaluate risk of bias, covering (a) bias due to confounding, (b) bias in participant selection, (c) bias in intervention classification, (d) bias due to deviations from intended interventions, (e) bias due to missing data, (f) bias in outcome measurement, and (g) bias in selection of reported results. Each domain was rated as low, moderate, or serious risk of bias. Studies were summarised as having low risk (low risk across all domains), moderate risk (low or moderate risk in all domains), or serious risk (serious risk in one or more domains).

The three authors independently conducted the RoB2 assessments, while two (TG and HP) independently performed the ROBINS-I assessments. Any grading disagreements were resolved through discussion until a consensus was reached on the final grading.

### Statement of equity, diversity and inclusion

All eligible studies were included in the systematic review regardless of participant characteristics such as sex, gender, race/ethnicity, socioeconomic status, or representation from marginalised groups, as these factors were not specified in our data analysis forms. The author group consists of two male and one female researcher, representing a combination of PhD candidate (one author) and senior researchers (two authors), all affiliated with a single academic institution in Sweden. Two authors have clinical backgrounds while one author has experience in exercise science.

### Registration

This systematic review has not been registered in PROSPERO because it has not been aimed to report health related outcomes and effects. In this review we aimed only to describe and report only training variables of selected and analysed intervention studies.

## Results

**Figure 1:**
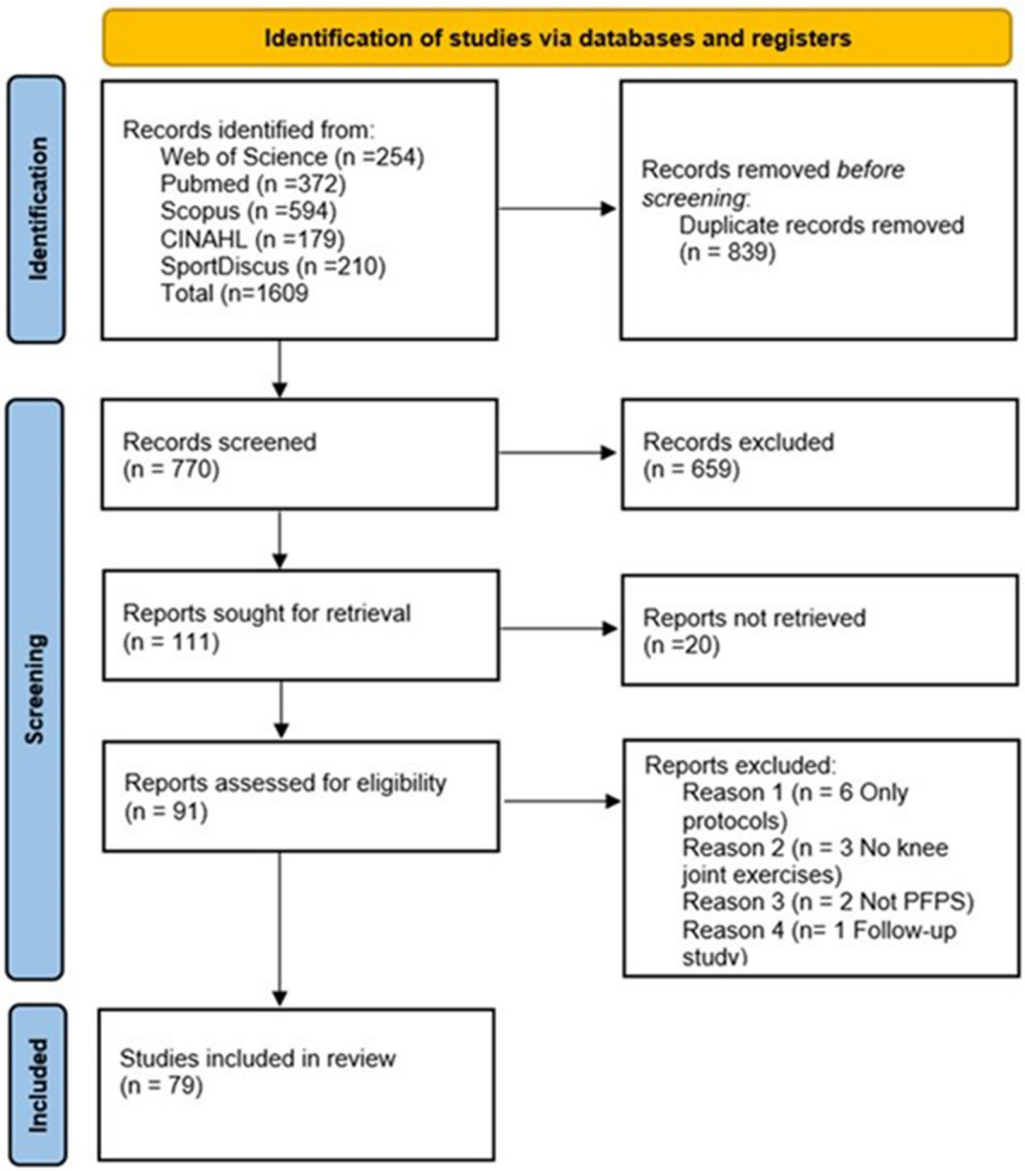
PRISMA flow diagram of the screening process.

A total of 1,609 studies were initially found through the search strategy. After screening and analysis, 79 studies (21–99) were deemed eligible by the inclusion criteria and included in this systematic review. The flow chart of this process can be seen in Figure 1.

### Exercises for the knee extensors

A total of 240 exercises for the knee muscles were identified in the included studies. The exercises used in the included studies could through naming, illustration, or description of how they were performed, be summarised to a total of 19 different types of exercises. As the majority of studies incorporated multiple exercises for knee extensor training, Table 1 provides a summary of their implementation. This includes the frequency of use for each exercise, and details on the number of repetitions and sets, ROM and training intensity. In summary, the most frequently used exercises were straight leg raises, squats, and open chain knee extension. The most common training structure consisted of three sets of ten repetitions using only body weight, without additional resistance.

**Table 1:** Descriptives of the knee extensor exercises, including their implementation.

Exercise	Used in studies (*n*)	Number of repetitions: median (min-max)	Number of sets: median (min-max)	ROM: median (min-max)^a^	Intensity: median (min-max)
Straight leg raises	33	10 (5–30)	3 (1–6)	Isometric 0°	BW (BW—75% 1 RM)
Squat	29	10 (5–25)	3 (1–4)	0–45° (0–90°)	BW (BW—80% 1 RM)
Knee extension (sitting)	28	10 (3–30)	3 (1–6)	90–45° (90–0°)	70% 1 RM (BW—80% 1 RM)
Terminal knee extension	26	10 (5–55)	3 (1–5)	10° (0–30°)	BW (BW—10 RM)
Wall squat	22	10 (10–20)	3 (1–3)	0–45° (0–90°)	BW (BW—BW + 10%)
Step downs	22	20–10	3 (1–3)	20 cm (3,6–25 cm)	BW (BW—BW + 10%)
Single leg squat	17	10 (6–20)	3 (1–3)	0–90° (0–90°)	BW (BW—70% 1 RM)
Quad Set	15	10 (10–15)	3 (1–3)	Isometric 0°	BW (BW—BW + Resistance band)
Leg press	13	10 (7–20)	3 (2–4)	0–45° (0–90°)	70% 1 RM (50%–80% 1 RM)
Lunges	13	10 (6–25)	3 (3–3)	0–90° (0–90°)	BW (BW—70% 1 RM)
Isometric contraction	10	10 (10–10)	3 (1–3)	0° (0–90°)	NA
Hip thrust	4	10 (6–20)	3 (2–3)	?	BW (BW—70% 1 RM)
Sit to stand	2	10 (6–20)	1 (1–1)	0–90° (0–90°)	BW
Cross-ski in water	1	2 min	2	?	NA
Frontal kick in the water	1	2 min	2	?	NA
Hopping activities	1	2 min	2	?	NA
Single leg step jump	1	15–10	3	?	BW
Single leg wall squat	1	60 s	3	0–60° (−)	BW
Wingate sprint	1	30 s	6	NA	1 kp

^a^ Degrees of knee flexion.

BW, body weight; ?, was not specified; NA, not applicable; 1RM, one repetition maximum; kp, kilopond; ROM, range of motion.

### Study characteristics

A total of 296 outcome measures (82 different) were identified in the included studies. To make this data comprehensible, the 82 different outcome measures used were synthesised and categorised into 9 different categories that can be seen in Table 2. The *Strength Tests* category included outcomes like isometric knee extensor strength and trunk endurance. The *Pain Questionnaires* category encompassed assessments such as the Anterior Knee Pain Scale (AKPS) and the PFP Severity Scale, whereas the *Pain* category included measures like the Visual Analog Scale (VAS) and the Numeric Pain Rating Scale. *Functional Tests* featured performance-based measures, including the single-leg hop and eccentric knee control tests, while *Functional Questionnaires* involved tools such as the Tegner Activity Scale. The *Anatomy Tests* category covered outcomes like M. Quadriceps length and muscle thickness, and *Movement Analysis* included various kinematic measures. The *Quality of Life Questionnaire* category featured instruments like Short Form Health Survey (SF-36), and the *Compliance* category included measures of feasibility and adherence.

**Table 2:** Descriptive characteristics of exercise protocol and outcomes the studies.

Type of training (*n* of the studies)		Outcomes measures used (*n* of the studies)		Progression used (*n* of the studies)		Duration: median (min-max)	Training frequency: median (min-max)
Strength training	(41)	Strength tests	(67)	Did not use progression	(52)	6 weeks (2^a^–21 weeks)	3 sessions/week (1 session/week- 2 sessions/day)
Exercise therapy	(14)	Pain questionnaire	(63)	Increased intensity	(12)		
Physiotherapy	(9)	Pain	(62)	Used multiple types of progression	(7)		
Conservative treatment	(7)	Functional tests	(45)	Increase volume (sets and reps)	(5)		
Functional stabilisation	(3)	Functional questionnaires	(27)	Increased range of motion	(2)		
Blood flow restriction	(1)	Anatomy tests	(17)	Progression of exercises	(1)		
Low load resistance	(1)	Movement analysis	(6)				
McConnel regime	(1)	Quality of life questionnaire	(5)				
Neurofeedback	(1)	Compliance	(4)				
Task specific	(1)						

^a^ 1 study was cross sectional for 1 session.

The authors categorised the types of training as described in the various interventions into ten distinct categories, with strength training being the most prevalent used in 41 of the 79 included studies. Additionally, method by which progression was implemented, the duration of the interventions, and the training frequency are presented in Table 2. A full description of the included studies regarding title, author, year, population, country, results, and setting can be found in Supplementary S2.

### Completeness of description

**Figure 2:**
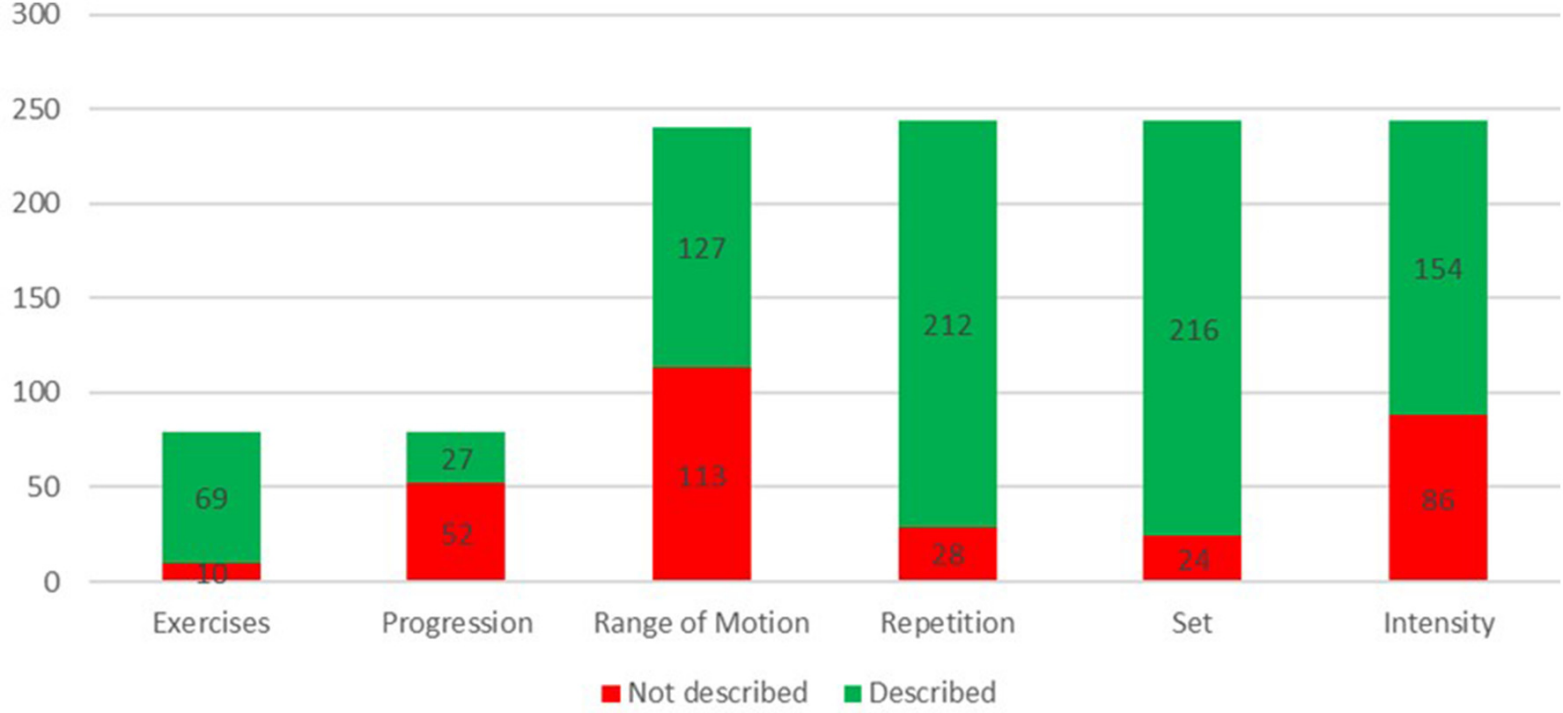
Reporting of exercise and training variables across studies.

Out of the 79 included studies, 25 provided comprehensive information on the data items investigated in this research. Details of the number of studies that specified the exercises used and whether they included descriptions of training progression are presented in Figure 2. Additionally, the figure indicates whether the ROM, number of repetitions and sets, and the load used were described for the total of 240 exercises extracted from the studies.

### Risk of bias

Out of the 79 included studies 61 where randomised controlled trials (RCT) and 18 were non-randomised. Of the 61 RCT assessed in regard of risk of bias, 28 exhibited some concerns while 33 raised high risk regarding potential bias. The overall risk of bias was largely consistent across the studies, with high risk primarily stemming from how outcomes were measured. In training interventions, when participants evaluate their own outcomes without blinding, it typically results in a high-risk rating in this area. Additionally, 30 of the 61 studies failed to clearly outline their randomisation process, and only 15 of the 61 had a preregistered study protocol in a clinical trial registry.

All 18 non-randomised studies included in this review had serious concerns regarding bias. When participants assessed outcomes, such as pain of function, without being blinded, it constituted a serious risk of bias in the measurement of outcomes domain. If this factor were not considered, 7 out of the 18 studies would still be classified as having a serious risk of bias, primarily due to confounding issues (5 out of 18).

A complete documentation of the Rob2 and ROBINS-I assessment can be found as Supplementary S3, S4.

## Discussion

The key findings from this study are: (1) the most common training regimen for knee extensor training involves the exercises straight leg raise, squat, and open chain knee extension, typically performed in three sets of ten repetitions using body weight as resistance, and (2) higher reporting standards for training interventions to improve clarity and applicability of training interventions to clinical practice are warranted. Despite limited descriptions of exercise execution, these findings can still provide clinicians with valuable guidance when prescribing knee extensor training for patients with PFP.

### Outcomes

The included studies utilised a total of 82 different outcomes. The most frequently used were VAS, AKPS, and knee extensor strength, appearing 54, 43, and 31 times, respectively. However, among the 82 different outcomes used in the included studies, the majority were only utilised once. This wide variation in outcomes poses a challenge for comparing and synthesizing results across studies, limiting the ability to draw clear conclusions and assess the effectiveness of interventions for PFP. Standardising outcomes ensures that the interventions effectively target and improve the intended qualities, and secondly, enables future meta-analyses and synthesis studies to accurately compare outcomes across different intervention studies (100).

### Strength training

The majority of the studies included in this review described their intervention as “strength training”. According to the American College of Sports Medicine, recommended strength training should be performed such as 8–12 repetitions, 2–4 sets with a resistance equivalent of 70%–100% of one repetition maximum (1RM) (101). An alternative to training at or above 70% of 1RM is to train close to muscular failure on lower loads, which also seems to positively affects strength (102). Most included studies used a training regime where the exercises were performed within this range regarding repetitions and sets. However, the intensity used in the different exercises, in relation to the number of repetitions performed, does not reflect the recommended training regime aimed to improve strength (101). If the purpose of the “strength training” interventions was to increase the strength of the participants, another training regime might be more suitable, where the load stands in relation to the given repetition and set scheme. When high-intensity training with fewer repetitions is not feasible, low-intensity training should include a sufficiently high number of repetitions to induce muscular fatigue, which is essential for stimulating strength adaptations (103).

Although the progression of training variables has a critical role in improving physical abilities (104), most of the included studies lacked or did not describe if a progression was used in the intervention. The ACSM emphasizes the importance of progressive overload to elicit continued strength gains (101). Without sufficient load and progression, the physiological stimulus required for strength development is likely inadequate. As such, several interventions labeled as “strength training” in the included studies may be more accurately described as low-intensity muscle activation or general exercise routines. This cautious approach, likely aimed at minimizing the risk of pain exacerbation, often resulted in conservative training volumes and intensities. However, if the goal is to improve strength while managing pain risk, the use of individualized progression or periodized loading models could offer a more effective and safer alternative. Gradual increases in training load allow for strength gains while potentially maintaining tolerability in populations with pain or physical limitations (105).

### Range of motion

ROM was the least reported training variable in this systematic review, despite its crucial role in muscle development and strength gains through enhanced muscle activation, mechanical tension, and stretch stimulus (106, 107). Furthermore, none of the included studies utilised a ROM greater than 90 degrees of knee flexion. This limitation was likely influenced by recommendations suggesting that PFP patients may benefit from limiting knee flexion to 45–60 degrees during closed-chain exercises to minimise patellofemoral joint forces and, consequently, reduce pain (12, 14, 15). These guidelines, however, are primarily based on biomechanical research conducted in healthy populations, leaving a gap in understanding the specific impact of larger ROM on patellofemoral joint stress among PFP patients. Further investigation is needed to establish whether the increased patellofemoral joint forces associated with higher ROM could have adverse effects on this population, emphasising the importance of evidence-based ROM guidelines that directly pertain to individuals with PFP.

### Limited reporting

This systematic review included 79 articles that evaluated the effectiveness of rehabilitation protocols for PFP, specifically through interventions involving knee extensor strengthening exercises. The results indicate that the exercise regimes were generally reported inadequately, lacking the necessary details for accurate replication in future studies. This finding is in accordance with Holden et al. in 2018 (10) and Yamato et al. (108) in 2016 which found that the exercise regimes used in interventions are often poorly reported. Only 25 out of 79 studies included in this review had a complete description of the training variables in their intervention. This limited reporting restricts the ability to provide thorough recommendations and treatment guidelines for practitioners. Enhanced transparency in reporting would enable more effective replication of successful protocols and facilitate the development of standardised treatment guidelines (109).

### Risk of bias

More than half of the 61 randomized controlled trials were rated as high risk of bias, primarily due to unblinded outcome assessment and poorly described randomisation methods, issues particularly relevant in studies relying on self-reported measures like pain and function. However, this review was designed as a descriptive analysis of exercise characteristics, not as an evaluation of intervention effectiveness. As such, while risk of bias affects the internal validity of individual studies, its impact on our primary aim—summarizing how knee extensor training has been implemented—is likely limited. Nevertheless, the consistent presence of bias underscores the need for higher-quality, transparently reported trials in this area.

### Study designs

This systematic review was aimed to summarise and describe the utilisation of knee extensor exercises within training interventions for PFP, and to do so comprehensively, we included both randomised and non-randomised studies. This inclusive approach allowed us to capture a broader spectrum of interventions, often incorporating additional treatments alongside knee extensor exercises, thereby providing a more complete picture of the current research landscape. However, because of the variability in study designs and the diversity of interventions included, it was impossible to quantitatively assess the separate effects of knee extensor exercises alone. Consequently, this review does not determine the comparative efficacy of different knee extensor exercises. Instead, it offers clinicians an overview of standard practices in knee extensor exercise prescriptions, which may inform and support clinical decision-making.

### Moving forward

Given that factors such as training volume, load, and ROM play a crucial role in promoting muscle hypertrophy and strength (102–104, 110), these variables should be carefully considered when designing training interventions, and detailed in research articles to improve the quality of patient treatment. To address these issues, future research should prioritize standardized reporting of exercise protocols, including clear definitions of exercise type and training parameters. Using structured frameworks and explicitly detailing prescription decisions can enhance transparency, improve study quality, and support more evidence-aligned clinical decision-making. Ultimately, better reporting will help bridge the gap between research and practice, enabling more tailored and informed rehabilitation strategies for individuals with PFP.

### Strengths and limitations

This is the first study of its kind to systematically describe which exercises and training variables researchers prescribed for the knee joint muscles as treatment for PFP. From these results, practitioners can find some support in which exercises, training volume, load, and ROM to use in treating of patients with PFP. However, some limitations in the present study need to be acknowledged. First, the findings of this study are limited to being descriptive in nature. They provide information on the exercises used and how they have been applied to treat patients with PFP. Consequently, since the effect sizes of the included studies and exercises have not been investigated, no conclusions can be drawn about whether one exercise or method of prescribing an exercise is superior to another. However, assessing effect sizes was beyond the intended aim of this research.

Secondly, even though the authors of this study have experience as clinicians and researchers and the expertise of librarians was used to find correct search terms and construct a search string, relevant studies might have been missed in the search for literature. Extensive efforts were made to ensure the search was as comprehensive as possible by systematically incorporating keywords, concepts, and terminology identified in prior studies and related reviews, but some relevant literature may still have been overlooked.

Thirdly, the initial screening and exclusion of studies by title was conducted solely by the first author, which may introduce a risk of selection bias. However, in cases where there was any uncertainty regarding a study's relevance, it was retained for further evaluation. Additionally, all abstracts were independently reviewed by all three authors to further assess relevance and remove non-eligible studies. This collaborative process likely mitigated the risk of bias and improved the reliability of study selection.

## Conclusion

In conclusion, this systematic review highlights common exercise regimes prescribed for PFP, primarily involving knee extensor exercises like straight leg raise, squat, and open-chain knee extension. However, key training variables, especially intensity and ROM, are insufficiently addressed, despite their importance for strength development and PFP-related pain. Enhancing transparency in reporting training variables would support more effective, evidence-based interventions and the development of standardised treatment guidelines for PFP. Furthermore, a notable discrepancy exists between the described interventions as strength training and the recommended load and volume parameters for optimal strength development. Despite these limitations, this review offers a clinically relevant overview of how knee extensor training is currently being implemented for PFP. While practitioners should be cautious about drawing conclusions regarding efficacy, the compiled data may still inform exercise selection and structuring decisions in clinical practice.

## Data Availability

The original contributions presented in the study are included in the article/Supplementary Material, further inquiries can be directed to the corresponding author.
