# Supplementary material for: Knee extensor training in patients with patellofemoral pain: a systematic review and synthesis
Source: Front Rehabil Sci. 2025 Aug 11;6:1641054. doi: 10.3389/fresc.2025.1641054 (PMC12377044; doi:10.3389/fresc.2025.1641054)
Supplement: Supplementary file 1 [file Supplementaryfile1.docx]

Supplement 1

Search strategies

Web of Science

|  | Search Query | Results |
| --- | --- | --- |
| 1 | 1: TS=("patellofemoral pain syndrome") | Results: 1341 |
| 2 | 2: TS=("patellofemoral joint pain") | Results: 40 |
| 3 | 3: TS=("anterior knee pain") | Results: 2746 |
| 4 | 4: TS=("resistance training") | Results: 15431 |
| 5 | 5: TS=("quadriceps musc*") | Results: 5902 |
| 6 | 6: TS=("quadriceps stren*") | Results: 3306 |
| 7 | 7: TS=("quadriceps exer*") | Results: 174 |
| 8 | 8: TS=("quadriceps resis*") | Results: 14 |
| 9 | 9: TS=("knee resis*") | Results: 7 |
| 10 | 10: TS=("knee stren*") | Results: 678 |
| 11 | 11: TS=("knee exer*") | Results: 123 |
| 12 | 12: TS=("knee musc*") | Results: 1029 |
| 13 | 13: TS=("plica syndrome") | Results: 121 |
| 14 | 14: #1 OR #2 OR #3 OR #13 | Results: 3820 |
| 15 | 15: #12 OR #11 OR #10 OR #9 OR #8 OR #7 OR #6 OR #5 OR #4 | Results: 25410 |
| 16 | 16: #15 AND #14 | Results: 394 |

Pubmed

| 1 | Search: "patellofemoral pain syndrome" [mh] Filters: Full text | 1,142 |
| --- | --- | --- |
| 2 | Search: "patellofemoral pain syndrome"[tiab] Filters: Full text | 791 |
| 3 | Search: "anterior knee pain"[tiab] Filters: Full text | 2,075 |
| 4 | Search: "patellofemoral joint pain"[tiab] Filters: Full text | 37 |
| 5 | Search: #1 OR #2 OR #3 OR #4 Filters: Full text | 3,243 |
| 6 | Search: "quadriceps muscl*"[mh] Filters: Full text | 6,702 |
| 7 | Search: "knee muscl*" Filters: Full text | 756 |
| 8 | Search: "quadriceps exercise*" Filters: Full text | 138 |
| 9 | Search: "quadriceps strength*" Filters: Full text | 2,143 |
| 10 | Search: "quadriceps resistan*" Filters: Full text | 13 |
| 11 | Search: "knee strength*" Filters: Full text | 518 |
| 12 | Search: "Knee exercis*" Filters: Full text | 90 |
| 13 | Search: #6 OR #7 OR #8 OR #8 OR #9 OR #10 OR #11 OR  #12 Filters: Full text | 9,27 |
| 14 | Search: "Prospective Studies"[Mesh] Filters: Full text | 607,913 |
| 15 | Search: "Follow-Up Studies"[Mesh] Filters: Full text | 552,968 |
| 16 | Search: "Cohort Studies"[Mesh] Filters: Full text | 2,224,490 |
| 17 | Search: "Non-Randomized Controlled Trials as  Topic"[Mesh] Filters: Full text | 1,053 |
| 18 | Search: "Randomized Controlled Trials as Topic"[Mesh] Filters: Full  text | 153,378 |
| 19 | Search: #14 OR #15 OR #16 OR #17 OR #18 Filters: Full text | 2,361,045 |
| 20 | Search: #5 AND #13 Filters: Full text | 334 |
| 21 | Search: #5 AND #13 AND #19 Filters: Full text | 62 |
| 22 | Search: #5 AND #13 NOT Review | 283 |

Sportdiscus

| **#** | **Query** | **Results** |
| --- | --- | --- |
| 1 | AB "patellofemoral pain syndrome" | 562 |
| 2 | AB "anterior knee pain" | 762 |
| 3 | AB "patellofemoral joint pain" | 24 |
| 4 | DE "plica syndrome" | 727 |
| 5 | DE "Resistance training" | 6,53 |
| 6 | DE "quadriceps muscle" | 3,366 |
| 7 | AB "knee muscle" | 260 |
| 8 | AB "knee muscl*" | 414 |
| 9 | AB "quadriceps exercise" | 36 |
| 10 | AB "quadriceps streng*" | 870 |
| 11 | AB "quadriceps resist*" | 8 |
| 12 | AB "knee streng*" | 271 |
| 13 | AB "knee exerci*" | 37 |
| 14 | (AB "knee exerci*") AND (S1 OR S2 OR S3 OR S4) | 5 |
| 15 | (S1 OR S2 OR S3 OR S4) | 1,71 |
| 16 | ((S1 OR S2 OR S3 OR S4)) AND (S5 OR S6 OR S7 OR S8 OR S9 OR S10 OR S11 OR S12 OR S13) | 213 |
| 17 | (S5 OR S6 OR S7 OR S8 OR S9 OR S10 OR S11 OR S12 OR S13) | 10,863 |

CINAHL

| **#** | **Query** | **Results** |
| --- | --- | --- |
| 1 | AB "patellofemoral joint pain" | 21 |
| 2 | AB "patellofemoral pain syndrome" | 563 |
| 3 | AB "anterior knee pain" | 989 |
| 4 | DE "plica syndrome" | 18 |
| 5 | DE "Resistance training" | 7,122 |
| 6 | DE "quadriceps muscle" | 0 |
| 7 | DE "quadriceps muscle" | 7,257 |
| 8 | AB "knee muscl*" | 408 |
| 9 | AB "quadriceps exercise" | 36 |
| 10 | AB "quadriceps streng*" | 1,216 |
| 11 | AB "quadriceps resist*" | 9 |
| 12 | AB "knee streng*" | 314 |
| 13 | AB "knee exerci*" | 42 |
| 14 | S1 OR S2 OR S3 OR S4 | 1,497 |
| 15 | S5 OR S6 OR S7 OR S8 OR S9 OR S10 OR S11 OR S12 OR S13 | 9,074 |
| 16 | S14 AND S15 | 99 |

Scopus

| 1 | TITLE-ABS-KEY ( "patellofemoral pain syndrome" ) | 2,429 document results |
| --- | --- | --- |
| 2 | TITLE-ABS-KEY ( "anterior knee pain" ) | 2,915 document results |
| 3 | TITLE-ABS-KEY ( "patellofemoral joint pain" ) | 51 document results |
| 4 | TITLE-ABS-KEY ( "plica syndrome" ) | 190 document results |
| 5 | TITLE-ABS-KEY ( "resistance traini*" ) | 31,178 document results |
| 6 | TITLE-ABS-KEY ( "quadriceps musc*" ) | 11,796 document results |
| 7 | TITLE-ABS-KEY ( "knee resis*" ) | 16 document results |
| 8 | TITLE-ABS-KEY ( "knee musc*" ) | 1,247 document results |
| 9 | TITLE-ABS-KEY ( "quadriceps exerc*" ) | 220 document results |
| 10 | TITLE-ABS-KEY ( "quadriceps stren*" ) | 2,512 document results |
| 11 | TITLE-ABS-KEY ( "quadriceps resis*" ) | 15 document results |
| 12 | TITLE-ABS-KEY ( "knee stren*" ) | 685 document results |
| 13 | TITLE-ABS-KEY ( "knee exer*" ) | 186 document results |
| 14 | ( TITLE-ABS-KEY ( "patellofemoral joint pain" ) ) OR ( TITLE-ABS-KEY ( "patellofemoral pain syndrome" ) ) OR ( TITLE-ABS-KEY ( "plica syndrome" ) ) OR ( TITLE-ABS-KEY ( "anterior knee pain" ) ) | 4,941 document results |
| 15 | ( TITLE-ABS-KEY ( "quadriceps stren*" ) ) OR ( TITLE-ABS-KEY ( "resistance traini*" ) ) OR ( TITLE-ABS-KEY ( "quadriceps musc*" ) ) OR ( TITLE-ABS-KEY ( "quadriceps exerc*" ) ) OR ( TITLE-ABS-KEY ( "quadriceps resis*" ) ) OR ( TITLE-ABS-KEY ( "knee musc*" ) ) OR ( TITLE-ABS-KEY ( "knee resis*" ) ) OR ( TITLE-ABS-KEY ( "knee exer*" ) ) OR ( TITLE-ABS-KEY ( "knee stren*" ) ) | 45,183 document results |
| 16 | ( ( TITLE-ABS-KEY ( "quadriceps stren*" ) ) OR ( TITLE-ABS-KEY ( "resistance traini*" ) ) OR ( TITLE-ABS-KEY ( "quadriceps musc*" ) ) OR ( TITLE-ABS-KEY ( "quadriceps exerc*" ) ) OR ( TITLE-ABS-KEY ( "quadriceps resis*" ) ) OR ( TITLE-ABS-KEY ( "knee musc*" ) ) OR ( TITLE-ABS-KEY ( "knee resis*" ) ) OR ( TITLE-ABS-KEY ( "knee exer*" ) ) OR ( TITLE-ABS-KEY ( "knee stren*" ) ) ) AND ( ( TITLE-ABS-KEY ( "patellofemoral joint pain" ) ) OR ( TITLE-ABS-KEY ( "patellofemoral pain syndrome" ) ) OR ( TITLE-ABS-KEY ( "plica syndrome" ) ) OR ( TITLE-ABS-KEY ( "anterior knee pain" ) ) ) | 594 document results |
