# Supplementary material for: Knee extensor training in patients with patellofemoral pain: a systematic review and synthesis
Source: Front Rehabil Sci. 2025 Aug 11;6:1641054. doi: 10.3389/fresc.2025.1641054 (PMC12377044; doi:10.3389/fresc.2025.1641054)
Supplement: Supplementary file 2 [file Supplementaryfile2.docx]

**Supplement 2.**

***Descriptive information on the included studies***

| **Nr.** | **Name** | **Author** | **Year** | **Duration** | **Population** | **Study design** | **Country** | **Setting** |
| --- | --- | --- | --- | --- | --- | --- | --- | --- |
| 1 | A controlled trial of weight-bearing versus non-weight-bearing exercises for patellofemoral pain [33] | Herrington, L. and A. Al-Sherhi | 2007 | 3 sessions/w x 6w | 45M 18-35y | RCT | Saudi arabia | hospital |
| 2 | A feasibility study of a novel, task-specific movement training intervention for women with patellofemoral pain [34] | Salsich, G. B., et al. | 2018 | 1x/day x 6w | 25F 18-36y | Feasability | USA | Physio Clinic |
| 3 | A proximal progressive resistance training program targeting strength and power is feasible in people with patellofemoral pain [35] | Barton, C. J., et al. | 2019 | 3 sessions/w x 12w | 6F 5M 28-38y | Feasability | Australia | Physio Clinic |
| 4 | Adding neuromuscular training to a strengthening program did not produce additional improvement in clinical or kinematic outcomes in women with patellofemoral pain: A blinded randomised controlled trial [36] | Silva, N. C., et al. | 2023 | 2x/w x 12w | 71F 18-25y | RCT | Brazil | biomechanic lab |
| 5 | Additional effects of an individualized risk factor-based approach on pain and the function of patients with patellofemoral pain syndrome: A randomized controlled trial [37] | Halabchi, F. Mazaheri, R. Mansournia, M. A. Hamedi, Z. | 2015 | 1x/day x 12w | 53M/F 18-40y | RCT | Iran | sport medicine clinic |
| 6 | An outcome study of chronic patellofemoral pain syndrome: Seven-year follow-up of patients in a randomized, controlled trial [38] | Kannus, P., et al. | 1999 | 1x/day x 6w | 28F 25M 18-36y | RCT | Finland | clinic |
| 7 | Anterior knee pain: A clinical comparison of rehabilitation methods [39] | Roush, M. B., et al. | 2000 | 2x/dayx12w | 38F 26M 20-35y | RCT | USA | home training, hospital |
| 8 | Anteromedial versus posterolateral hip musculature strengthening with dose-controlled in women with patellofemoral pain: A randomized controlled trial [40] | Almeida, G. P. L., et al. | 2021 | 2 session/w x 6v | 52F 18-35y | RCT | Brazil | Physio Clinic |
| 9 | Biofeedback supplementation to physiotherapy exercise programme for rehabilitation of patellofemoral pain syndrome: A randomized controlled pilot study [41] | Yip, S. L. M. and G. Y. F. Ng | 2006 | 1 x/day x 8w | 16F 10M 22-55y | RCT | China | home training, hospital |
| 10 | Carryover effect of hip and knee exercises program on functional performance in individuals with patellofemoral pain syndrome [42] | Hamada, H. A., et al. | 2017 | 3 sessions/w x 4w | 30 F/M 18-29y | crossover randomized | Egypt | clinic |
| 11 | Closed Kinetic Chain exercises with or without additional hip strengthening exercises in management of Patellofemoral pain syndrome: A randomized controlled trial [43] | Ismail, M. M., et al. | 2013 | 3 sessions/w x 6w | 23F 9M 18-30y | RCT | Egypt | Physio Clinic |
| 12 | Comparing hip and knee focused exercises versus hip and knee focused exercises with the use of blood flow restriction training in adults with patellofemoral pain [44] | Constantinou, A., et al. | 2022 | 3 seesions/w x 4w | 33F 27F 18-40y 33 | RCT | Cypern | group Physio Clinic |
| 13 | Comparison between Mc connell patellar taping and conventional physiotherapy treatment in the management of patellofemoral pain syndrome - A randomised controlled trial [45] | Verma, C. and V. Krishnan | 2012 | 5x/day x 2w | 20F/M 18-35y | RCT | India | hospital |
| 14 | Could hip and knee muscle strengthening alter the pain intensity in patellofemoral pain syndrome? [46] | Razeghi, M., et al. | 2010 | 3x/w x 6w | 32F 18-30y | RCT | Iran | Physio Clinic |
| 15 | Disability in patients with chronic patellofemoral pain syndrome: A randomised controlled trial of VMO selective training versus general quadriceps strengthening [47] | Syme, G., et al. | 2009 | 8w | 41F, 28M | RCT | Scotland | supervised |
| 16 | Effect of Adding Kinesio Taping to Exercise Therapy in the Treatment of Patellofemoral Pain Syndrome [48] | Lee, J. H., et al. | 2023 | 2 sessions/day x 4v | 31F 8M 20-35 y | Retrospecitve comparative | South Korea | sport medicine center |
| 17 | Effect of eccentric isotonic quadriceps muscle exercises on patellofemoral pain syndrome: An exploratory pilot study [49] | Eapen, C., et al. | 2011 | 3 sessions/w x 2w | 12 F 8M,21-25y | Explorative pilot study | India | hospital |
| 18 | Effect of instrument-assisted soft tissue mobilization combined with blood flow restriction training on function, pain and strength of patients with patellofemoral joint pain [50] | Liu, Y. and L. Q. Wu | 2023 | 2x/w x 4w | 10F 16M 20-24y | RCT | China | supervised |
| 19 | Effect of Intraarticular Glycosaminoglycan Polysulfate Treatment on Patellofemoral Pain Syndrome - A Prospective, Randomized Double-Blind Trial Comparing Glycosaminoglycan Polysulfate With Placebo and Quadriceps Muscle Exercises [51] | Kannus, P., et al. | 1992 | 7 sessions/w x 6v | 28F 25M 18-36y | RCT | Finland | home training, hospital |
| 20 | Effect of muscle strengthening on perceived pain and static knee angles in young subjects with patellofemoral pain syndrome [52] | Villafañe, J. H., et al. | 2019 | 10sessions/3w | 10F 15-21y | Pilot cohort study | Italy | Physio Clinic |
| 21 | Effect of Weight and Non-Weight Bearing Quadriceps Exercises Versus Weight-Bearing Quadriceps Exercises on Patellofemoral Pain, Quadriceps Strength and Functional Ability: A Randomized Clinical Trial [53] | Naidu, S. K. and V. Kage | 2018 | 3x/w x 6w | 34F/M 18-42y | RCT | India | Clinic |
| 22 | Effectiveness of Isolated Hip Exercise, Knee Exercise, or Free Physical Activity for Patellofemoral Pain: A Randomized Controlled Trial [54] | Hott, A., et al. | 2019 | 3 sessions/w x 6w | 73F 37M 16-40y | RCT | Norway | Physio Clinic |
| 23 | Effectiveness of static quadriceps stretching in individuals with patellofemoral joint pain [55] | Peeler, J. and J. E. Anderson | 2007 | 1x/day 8w | 54F 29M 18-45y | Control | Canada | home training |
| 24 | Effectiveness of tele-prescription of therapeutic physical exercise in patellofemoral pain syndrome during the COVID-19 pandemic [56] | Albornoz-Cabello, M., et al. | 2021 | 3 sessions/w x 4w | 28F 26M 25-67 | RCT | Spain | tele-presciption |
| 25 | Effects of functional stabilization training on pain, function, and lower extremity biomechanics in women with patellofemoral pain: A randomized clinical trial [57] | De Marche Baldon, R. | 2014 | 3 sessions/w x 8w | 31F 18-30y | RCT | Brazil | Physio Clinic |
| 26 | Effects of High-Intensity Aquatic or Bicycling Training in Athletes with Unilateral Patellofemoral Pain Syndrome [58] | Fang, B., et al. | 2022 | 3 sessions/w x 8w | 54F/M 18-22y | Experimental | South Korea | Clinic |
| 27 | Effects of hip and hip core muscles strengthening versus knee muscle strengthening for the management of anterior knee pain [59] | Ali, L., et al. | 2021 | 5 serssion/w x 6v | 21F 9M 15-35y | Quasi experimental | Pakistan | Physio Clinic |
| 28 | Effects of McConnell taping combined with strengthening exercises of vastus medialis oblique in females with patellofemoral pain syndrome [60] | Begum, R., et al. | 2020 | 4-5 seesion/w x 2v | 51F 27-43y | Cohort | Pakistan | Clinic |
| 29 | Effects of Radiofrequency Diathermy Plus Therapeutic Exercises on Pain and Functionality of Patients with Patellofemoral Pain Syndrome: A Randomized Controlled Trial [61] | Albornoz-Cabello, M., et al. | 2023 | 7 session/W x 3v | 29F 27M 38-48y | RCT | Spain | Physio Clinic |
| 30 | Effects of Static and Dynamic Stretching With Strengthening Exercises in Patients With Patellofemoral Pain Who Have Inflexible Hamstrings: A Randomized Controlled Trial [62] | Lee, J. H., et al. | 2021 | 2 sessions/day x 12v | 27 F19M 17-34y | RCT | South Korea | Physio Clinic |
| 31 | Effects of tibiofemoral mobilization in patients of Patellofemoral pain syndrome [63] | Fatimah, I. and S. Waqqar | 2021 | 3 sessions/w x 4w | 52F/M 25-35y | RCT | Pakistan | hospital |
| 32 | Effects of whole body vibration training on isokinetic muscular performance, pain, function, and quality of life in female patients with patellofemoralpain: a randomized controlled trial [64] | Corum, M., et al. | 2018 | 3 seesions/w x 8w | 40F 25-40y | RCT | Turkey | supervised |
| 33 | Effects of whole-body vibration plus hip-knee muscle strengthening training on adult patellofemoral pain syndrome: a randomized controlled trial [65] | Wu, Z., et al. | 2022 | 3x/w x 6w | 17F 19M 25-30y | RCT | China | Physio Clinic |
| 34 | Efficacy of electromyographic-biofeedback supplementation training with patellar taping on quadriceps strengthening in patellofemoral pain syndrome among young adult male athletes [66] | Alonazi, A., et al. | 2021 | 5 session/w x 4v | 60M 22-29y | RCT | Saudi arabia | rehab center |
| 35 | Efficacy of high-power laser in alleviating pain and improving function of patients with patellofemoral pain syndrome: A single-blind randomized controlled trial [67] | Nouri, F., et al. | 2019 | 3x/day x 12w | 28F 12M 25-38y | RCT | Iran | hospital |
| 36 | Electrical stimulation therapies for active duty military with patellofemoral pain syndrome: A randomized trial [68] | Talbot, L. A., et al. | 2020 | 9w | 29F 101M 20-33y | RCT | USA | home training, Clinic |
| 37 | Evaluating Eccentric Hip Torque and Trunk Endurance as Mediators of Changes in Lower Limb and Trunk Kinematics in Response to Functional Stabilization Training in Women With Patellofemoral Pain [69] | Baldon, R. D., et al. | 2015 | 3 sessions/w x 8v | 31F 18-30y | RCT | Brazil | Physio Clinic |
| 38 | Feedback Leads to Better Exercise Quality in Adolescents with Patellofemoral Pain [70] | Riel, H., et al. | 2018 | 3x/w x 6w | 35F 5M 15-19y | RCT | Denmark | Physio Clinic |
| 39 | Foot orthoses and physiotherapy in the treatment of patellofemoral pain syndrome: randomised clinical trial [71] | Collins, N., et al. | 2009 | 14 sessions/w x 6w | 100F 79M 18-40y | RCT | Australia | supervised |
| 40 | Hip Posterolateral Musculature Strengthening in Sedentary Women With Patellofemoral Pain Syndrome: A Randomized Controlled Clinical Trial With 1-Year Follow-up [72] | Fukuda, T. Y., et al. | 2012 | 3 sessions/w x 4w | 54F 20-40y | RCT | Brazil | supervised |
| 41 | Hip-Strengthening Exercises Before Functional Exercises Reduced Pain in Women with Patellofemoral Pain Syndrome [73] | Dolak, K., et al. | 2012 | 3 sessions/w x 8w | 35F 16-35y | RCT | USA | Physio Clinic |
| 42 | Home-based exercise and patellar bracing in the treatment of patellofemoral pain syndrome [74] | Evcik, D., et al. | 2010 | 5 session/w x 6 w | 72F 14M 17-80y | RCT | Turkey | rehab center |
| 43 | Home-based exercise program and Health education in patients with patellofemoral pain: a randomized controlled trial [75] | Hong, Q. M., et al. | 2023 | 3 sessions/w x 6w | 17F 21M, 26-40y | RCT | China | home training |
| 44 | How effective is an evidence-based exercise intervention in individuals with patellofemoral pain? [76] | Greaves, H., et al. | 2021 | 6w | 7F 9M 22-43y | obervational | England | clinical |
| 45 | Individualized Physiotherapy in the Treatment of Patellofemoral Pain [77] | Keays, S. L., et al. | 2015 | 4w | 26F 15M 13-82y | RCT | Australia | Physio Clinic |
| 46 | Is hip strengthening the best treatment option for females with patellofemoral pain? A randomized controlled trial of three different types of exercises [78] | Saad, M. C., et al. | 2018 | 2x/w x 8w | 40F 18-28y | RCT | Brazil | Physio Clinic |
| 47 | Long-term efficacy of a short period of taping followed by an exercise program in a cohort of patients with patellofemoral pain syndrome [79] | Paoloni, M., et al. | 2012 | 3-4x/day x 9w | 29F 15M 18-38y | Cohort study | Finland | Physio Clinic |
| 48 | Low load resistance training with blood flow restriction decreases anterior knee pain more than resistance training alone. A pilot randomised controlled trial [80] | Korakakis, V., et al. | 2018 | 1 time | 40M 23-37y | RCT | Qatar | Physio clinic |
| 49 | Mechanical response of hip and knee muscles following randomized crossover trials in patellofemoral pain syndrome [81] | Hamada, H. A., et al. | 2020 | 3-4x/w x 3w | 17F 7 M 18-35y | RCT crossover | Egypt | Physio Clinic |
| 50 | Mobilization-with-movement prior to exercise provides early pain and functionality improvements in patients with patellofemoral pain syndrome [82] | Zemadanis, K., et al. | 2015 | 3x/w x 6w | 25F 15M 18-40y | RCT | Greece | supervised |
| 51 | Outcomes of a Weight-Bearing Rehabilitation Program for Patients Diagnosed With Patellofemoral Pain Syndrome [83] | Boling, M. C., et al. | 2006 | 3 session/w x 6w | 9F 5M 18-42y | Intervention | USA | Musculoskeletal research laboratory |
| 52 | Pain, Function, and Strength Outcomes for Males and Females With Patellofemoral Pain Who Participate in Either a Hip/Core- or Knee-Based Rehabilitation Program [84] | Bolgla, L. A., et al. | 2016 | 3 sessions/w x 6w | 124F 61M 18-35y | RCT | USA | rehab center |
| 53 | Patellofemoral pain: One year results of a randomized trial comparing hip exercise, knee exercise, or free activity [85] | Hott, A., et al. | 2020 | 3 sessions/w x 6w | 73F 39M 16-40y | RCT | Norway | rehab hospital |
| 54 | Physical therapy alters recruitment of the vasti in patellofemoral pain syndrome [86] | Cowan, S. M., et al. | 2002 | 1 session/w x 6w | 42F 23M 18-40y | RCT | Australia | supervised |
| 55 | Physiotherapeutic interventions on quadriceps muscle architecture in patello-femoral pain syndrome [87] | Mv, V. K., et al. | 2023 | 3x/w x 10w | 150F/M 18-40y | RCT | India | Physio Clinic |
| 56 | Physiotherapy for anterior knee pain: A randomised controlled trial [88] | Clark, D. I., et al. | 2000 | 7 sessions/w x 12w | 35F 45M 15-40y | RCT | England | Physio Clinic |
| 57 | Physiotherapy with and without superficial dry needling affects pain and muscle strength in patients with patellofemoral pain syndrome [89] | Abyaneh, H. M., et al. | 2016 | 5 sessions/w x 2w | 34F/M 18-50y | RCT | Iran | Physio Clinic |
| 58 | Posterlateral Hip Muscle Strengthening Versus Quadriceps Strengthening for Patellofemoral Pain: A Comparative Control Trial [90] | Khayambashi, K., et al. | 2014 | 3x/w x 8w | 18F 18M 20-35y | Comparative control trial | Iran | rehab facicilty |
| 59 | Quadriceps strengthening with and without blood flow restriction in the treatment of patellofemoral pain: A double-blind randomised trial [91] | Giles, L., et al. | 2017 | 3 sessions/w x 8w | 43F 36M 18-40y | RCT | New Zealand | physcio clinic |
| 60 | Short-Term Effects of Hip Abductors and Lateral Rotators Strengthening in Females With Patellofemoral Pain Syndrome: A Randomized Controlled Clinical Trial [92] | Fukuda, T. Y., et al. | 2010 | 3 sessions/w x 4w | 70F 20-40y | RCT | Brazil | supervised |
| 61 | Static and Dynamic Quadriceps Stretching Exercises in Patients With Patellofemoral Pain: A Randomized Controlled Trial [93] | Lee, J. H., et al. | 2021 | 2 sessions/day x 21w | 29F 15M 20-32y | RCT | South Korea | Physio Clinic |
| 62 | Strengthening of the hip and core versus knee muscles for the treatment of patellofemoral pain: A multicenter randomized controlled trial [94] | Ferber, R., et al. | 2015 | 3 sessions/w x 6w | 133F 66M 22-36y | RCT | USA, Canada | clinical research laboratories |
| 63 | Supervised exercise therapy versus usual care for patellofemoral pain syndrome: an open label randomised controlled trial [95] | van Linschoten, R., et al. | 2010 | 13w | 84F 47M 14-40y | RCT | Netherlands | General practice and sport physician practice |
| 64 | The Effect of Adding Specific Hip Strengthening Exercises to Conventional Knee Exercises in Patients With Patello-Femoral Pain Syndrome [96] | Telles, G., et al. | 2016 | 3-4x/w x 4v | 30F/M 15-30y | RCT | India | supervised |
| 65 | The effect of additional strengthening of hip abductor and lateral rotator muscles in patellofemoral pain syndrome: a randomized controlled pilot study [97] | Monika, R., et al. | 2016 | 5x/w x 6w | 10F 4M 17-40y | RCT pilot | Brazil | Clinical setting with home programme |
| 66 | The effect of hip and knee exercises on pain, function, and strength in patients with patellofemoral pain syndrome: A randomized controlled trial [98] | Nakagawa, T. H., et al. | 2008 | 2x/day x 6w | 55F 20-45y | RCT | Turkey | Department of Physical Medicine and Rehabilitation |
| 67 | The effect of postural stabilization exercises on pain and function in females with patellofemoral pain syndrome [99] | Şahin, M., et al. | 2016 | 3x/day x 6w | 42F 40-50y | RCT | Turkey | Physio Clinic |
| 68 | The Effect of Taping, Quadriceps Strengthening and Stretching Prescribed Separately or Combined on Patellofemoral Pain [100] | Yılmaz Yelvar, G. D., et al. | 2015 | 3x/day x 2w | 26F 15M 13-82y | RCT | Australia | Physio Clinic |
| 69 | The Effect of Twelve-w Neurofeedback Training on Pain, Proprioception, Strength and Postural Balance in Men with Patellofemoral Pain Syndrome: A Double-Blind Randomized Control Trial [101] | Mason, M., et al. | 2011 | 3 sessioner x 12w x 30 minuter | 32M 18-35y | RCT | Iran | rehab labarotory |
| 70 | The effectiveness of exercise therapy based on sahrmann approach in patients with patella-femoral pain syndrome [102] | Ahmadi, M., et al. | 2020 | 6ws | 30M/F 20-55y | RCT | Iran | University of Medical Sciences |
| 71 | The effectiveness of superimposed neuromuscular electrical stimulation combined with strengthening exercises on patellofemoral pain: A randomized controlled pilot trial [103] | Celik, D., et al. | 2020 | 3 session/w x 6w | 15F 12M 20-60y | RCT pilot | Turkey | supervised |
| 72 | The effects of a multimodal rehabilitation program on symptoms and ground-reaction forces in runners with patellofemoral pain syndrome [104] | Esculier, J. F., et al. | 2016 | 3 session/w x 8w | 21M/F 18-45y | Quasi-experimental | Canada | Gait-analysis laboratory and private physical therapy clinic |
| 73 | The effects of quadriceps strengthening on pain, function, and patellofemoral joint contact area in persons with patellofemoral pain [105] | Chiu, J. K. W., et al. | 2012 | 3x/w x 8w | 5F 4M 18-45y | Independent group comparison | China | supervised |
| 74 | The efficacy of treatment of different intervention programs for patellofemoral pain syndrome--a single blinded randomized clinical trial. Pilot study [106] | Avraham, F., et al. | 2007 | 6 session/w x3v | 42F/M 35y | RCT | Israel | clinnic |
| 75 | The McConnell regimen versus isometric quadriceps exercises in the management of anterior knee pain. A randomised prospective controlled trial [107] | Eburne, J. and G. Bannister | 1996 | 13w | 50M/F y? | RCT | England | clinic |
| 76 | The role of high voltage electrical stimulation in the rehabilitation of patellofemoral pain [108] | Akarcali, I., et al. | 2002 | 5 serssion/w x 5w | 44F/M 27-50y | cohort | Turkey | rehabilitaiton |
| 77 | Therapeutic effect of two muscle strengthening programs in patients with patellofemoral pain syndrome. A randomized controlled clinical trial [109] | Prieto-García, L. F., et al. | 2021 | 8w | 36F 4M 15-40y | RCT | Colombia | Physical Rehabilitation Center |
| 78 | Three-Year Outcome after a 1-Month Physiotherapy Program of Local and Individualized Global Treatment for Patellofemoral Pain Followed by Self-Management [110] | Keays, S. L., et al. | 2016 | 3x/day x 4w? | 23F 14M 13-82y | Prospective cohort study | Australia | Physio Clinic |
| 79 | Which factors predict outcome in the nonoperative treatment of patellofemoral pain syndrome? A prospective follow-up study [111] | Kannus, P. and S. Niittymaki | 1994 | 1x/day x 6w | 27F 22M 18-36y | Prospective follow up study | Finland | home training |
