# Supplementary figures and images for: Knee extensor training in patients with patellofemoral pain: a systematic review and synthesis
Source: Front Rehabil Sci. 2025 Aug 11;6:1641054. doi: 10.3389/fresc.2025.1641054 (PMC12377044; doi:10.3389/fresc.2025.1641054)

**Supplement 3**

**
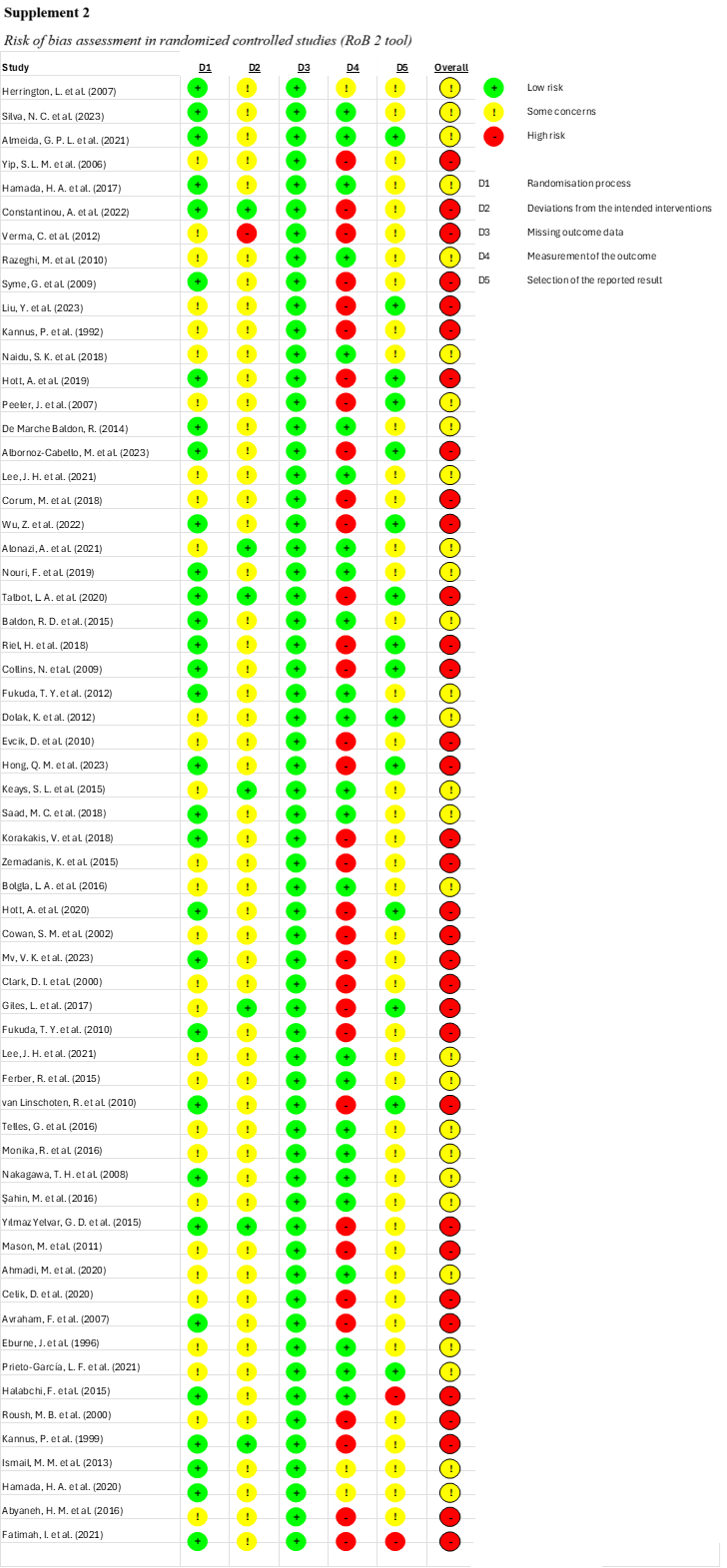
**

Supplement: Supplementary file 3 [file Supplementaryfile3.docx]

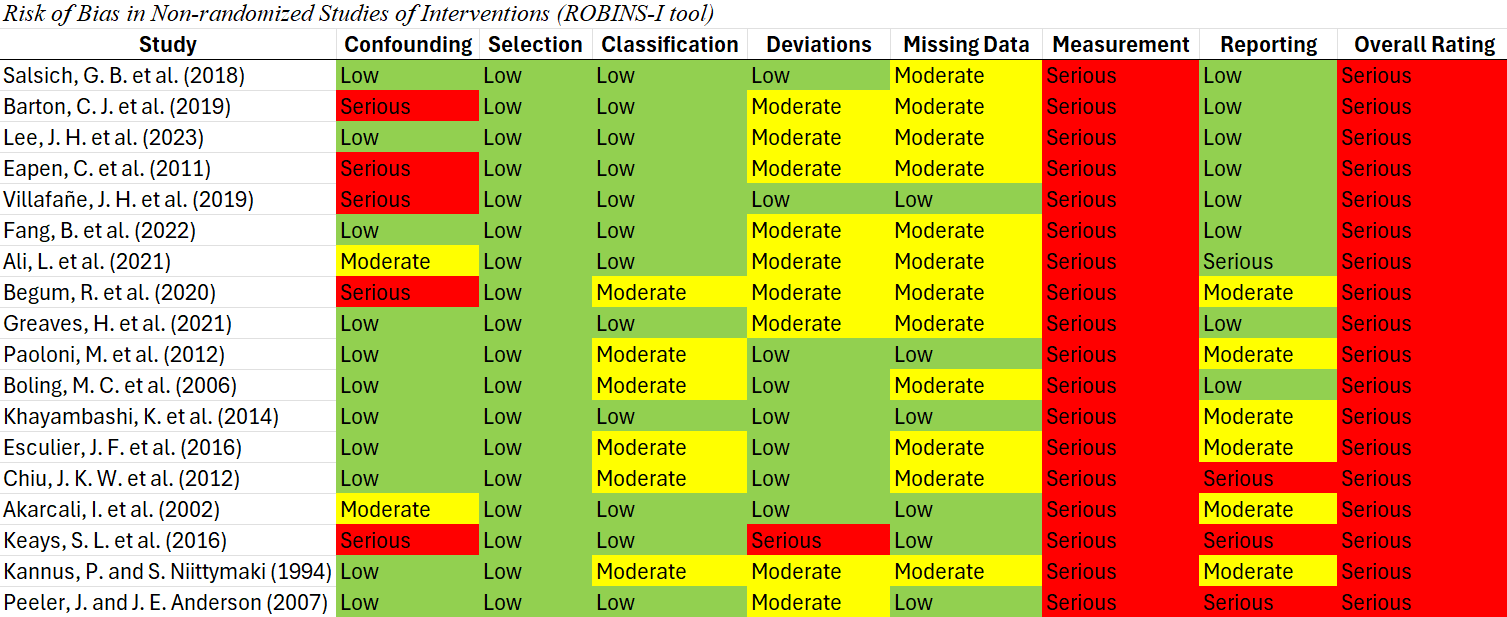
**Supplement 4**

Supplement: Supplementary file 4 [file Supplementaryfile4.docx]
